# Immune, Inflammatory, and Oxidative Responses in Cardiovascular Complications

**DOI:** 10.1155/2016/6858402

**Published:** 2016-03-28

**Authors:** Kota V. Ramana, Sanjay Srivastava, Aramati B. M. Reddy

**Affiliations:** ^1^Department of Biochemistry and Molecular Biology, University of Texas Medical Branch, Galveston, TX 77555, USA; ^2^Institute of Molecular Cardiology, University of Louisville, Louisville, KY 40202, USA; ^3^Department of Animal Biology, School of Life Sciences, University of Hyderabad, Hyderabad 500046, India

Cardiovascular diseases (CVD) are the leading causes of morbidity and mortality worldwide. Although treatment for risk factors for CVD such as hypertension, hyperlipidaemia, obesity, and diabetes has steadily decreased mortality due to cardiovascular events in the last three decades, the underling mechanisms for several CVD remain elusive. It is well known that oxidative stress and inflammation have been implicated in the etiology of several CVD. Further, oxidative stress and inflammation triggered signals can either directly cause injury to the cardiac tissues or increase the atherosclerotic process. However, most of the data for these studies are derived from experimental animals. Long term prospective clinical trials are required to validate the contribution of oxidative stress and inflammation in CVD. Moreover, better understanding of the signaling mechanisms that interplay between oxidative stress and inflammatory signaling would be beneficial in designing more selective and targeted therapy to combat these deleterious mediators of CVD. The purpose of this special issue is to assemble information from preclinical as well as clinical studies in order to provide an additional opportunity to identify the potential molecules/mechanisms that disrupt equilibrium between immune, inflammatory, and oxidative responses involved in the pathogenesis of CVD.

To better understand the pathogenesis of CVD in chronic kidney disease (CKD) P. Bartnicki et al. investigated the role of continuous erythropoietin receptor activator (CERA) on selective biomarkers of CVD. In this study, blood samples from 25 CKD patients with CERA treatment and 20 healthy subjects were evaluated for various inflammatory markers, biochemical parameters, and other endothelial specific biomarkers. Their results indicate that biomarkers of inflammation as well as endothelial dysfunction are significantly elevated in the nondialyzed CKD patients. Specifically, they found that biomarkers such as TNFr1, sICAM-1, and MMP9 could be the risk factors of CVD in CKD patients. Further, this study indicates that CERA treatment by using MPG-EPO diminished endothelial dysfunction and improves left ventricular function in CKD patients.

Another research article by B. Al-Shammari et al. investigated the pulmonary toxicity associated with the amiodarone, a drug used for treating ventricular and supraventricular dysrhythmia. In this study, treatment of rats with amiodarone leads to a time-dependent toxicity in the lungs. Specifically, amiodarone decreased antioxidant enzymes such as catalase, SOD, GPx, and GR and increased oxidative stress. Further, long term treatment of amiodarone for 3 to 4 weeks leads to significant toxicity as determined by granulomatous inflammation and interstitial pneumonitis. These studies indicate that imbalance of oxidative stress and antioxidant status and resulting inflammatory response could lead to pulmonary toxicity with amiodarone treatment.

Studies by Y. Cheng et al. reported the cardioprotective potential of formononetin, a natural plant derived isoflavone from Radix Astragali, using human cardiomyocyte H9c2 cells. Their results suggest that formononetin could protect cardiomyocytes from ischemia reperfusion injury by reducing the oxidative stress and inhibiting the mitochondrial permeability transition pore (mPTP) opening. Specifically, they have shown that formononetin induces AKT and GSK-3b phosphorylation leading to mPTP opening. These results also suggest that PI3K and PKC inhibitors could restore effects of formononetin-induced mPTP opening. Thus, these studies report that formononetin could be developed as a cardioprotective agent against acute myocardial infarction.

Another study by G. Zhang et al. examined the antioxidant effect of simvastatin on high-cholesterol diet-induced oxidative stress injury in aorta and hippocampus of rabbits. Their results suggest lipid peroxidation products as biomarkers of oxidative stress in high-cholesterol induced brain injury in rabbits. They have shown that simvastatin enhances antioxidant status (GSH-Px) in atherosclerotic aorta and decreases oxidative stress (malondialdehyde). This study again addresses the significance of oxidative and antioxidant imbalance in high-cholesterol induced tissue injury.

As an endnote, it is obvious from the recently published studies and current special issue papers that oxidative, immune, and inflammatory responses play a major role in the pathophysiology of cardiovascular complications. Therefore, the molecules that interrupt or neutralize the effects of oxidative, immune, and inflammatory responses could be the next important targets for the future drug discovery studies in CVD.

